# Does neighborhood environment influence girls' pubertal onset? findings from a cohort study

**DOI:** 10.1186/1471-2431-12-27

**Published:** 2012-03-13

**Authors:** Julianna Deardorff, Molly Fyfe, J Paul Ekwaru, Lawrence H Kushi, Louise C Greenspan, Irene H Yen

**Affiliations:** 1 School of Public Health, University of California, Berkeley, CA, USA; 2 Division of Research, Kaiser Permanente Northern California, Oakland, CA, USA; 3 School of Medicine, University of California, San Francisco, CA, USA

## Abstract

**Background:**

Pubertal onset occurs earlier than in the past among U.S. girls. Early onset is associated with numerous deleterious outcomes across the life course, including overweight, breast cancer and cardiovascular health. Increases in childhood overweight have been implicated as a key reason for this secular trend. Scarce research, however, has examined how neighborhood environment may influence overweight and, in turn, pubertal timing. The current study prospectively examined associations between neighborhood environment and timing of pubertal onset in a multi-ethnic cohort of girls. Body mass index (BMI) was examined as a mediator of these associations.

**Methods:**

Participants were 213 girls, 6-8 years old at baseline, in an on-going longitudinal study. The current report is based on 5 time points (baseline and 4 annual follow-up visits). Neighborhood environment, assessed at baseline, used direct observation. Tanner stage and anthropometry were assessed annually in clinic. Survival analysis was utilized to investigate the influence of neighborhood factors on breast and pubic hair onset, with BMI as a mediator. We also examined the modifying role of girls' ethnicity.

**Results:**

When adjusting for income, one neighborhood factor (Recreation) predicted delayed onset of breast and pubic hair development, but only for African American girls. BMI did not mediate the association between Recreation and pubertal onset; however, these associations persisted when BMI was included in the models.

**Conclusions:**

For African American girls, but not girls from other ethnic groups, neighborhood availability of recreational outlets was associated with onset of breast and pubic hair. Given the documented risk for early puberty among African American girls, these findings have important potential implications for public health interventions related to timing of puberty and related health outcomes in adolescence and adulthood.

## Background

Early puberty is associated with numerous negative mental health and physical health outcomes over the life course for girls and women, including obesity, type II diabetes, depression, conduct problems, substance use, teen pregnancy, and breast and other reproductive cancers [1-7]. Timing of pubertal onset among girls varies widely, with secondary sexual characteristics - the first observable signs of puberty - usually appearing around ages 10 to 11 years [8,9]. However, epidemiologic evidence confirms that certain pubertal markers are occurring earlier among girls in the U.S. than in the past, particularly onset of breast and pubic hair development [8,10-12]. In addition, there are marked disparities in pubertal timing across ethnic groups. Recent epidemiological research shows that at age 8 years approximately 43% of black girls, 31% of Hispanic girls and 18% of white girls have experienced onset of breast development [13].

This documented trend of earlier pubertal onset has prompted a cascade of research focused on potential antecedents that may explain variability in girls' timing of puberty. A variety of environmental and genetic factors have been identified that influence pubertal timing [14] with a recent enhanced focus on behavioral and environmentally-related factors, including overweight and obesity [15], family stressors [16-19], and environmental toxins [20] as potentially important determinants. No known studies, however, have examined the influence of neighborhood factors, nor how neighborhood effects might operate through girls' overweight, to influence pubertal onset. The current study addresses these gaps in the scientific literature.

Neighborhoods are complex entities encompassing culture, economics, history, governance, and the built and natural environments [21]. The neighborhoods in which children live have been shown to influence their behaviors and health outcomes [22-27]. There are conceivably two potential pathways through which a neighborhood environment might influence a girl's pubertal timing: (1) through reduced access to healthful foods and physical activity, which may lead to overweight and, in turn, earlier pubertal onset, and (2) through exposure to acute and chronic stress, which may trigger hormonal stress responses that prompt pubertal onset. An extensive body of literature supports the first proposed pathway. Research shows that body composition and physical activity levels are influenced by neighborhood environments and are also related to timing of girls' pubertal development [25,27-33]. Limited availability of fresh foods and poor access to safe recreational outlets may negatively influence girls' eating habits and physical activity patterns, which can lead to overweight and increased adiposity. In turn, hormonal changes related to increases in adiposity can subsequently accelerate pubertal maturation [34]. In addition, ample research suggests that exposure to stressful environmental conditions, particularly within the family realm, confers risk for earlier pubertal development [14] ostensibly by influencing hormonal pathways that trigger puberty [19]. As such, neighborhood stressors may operate in a similar manner to stress in the family and directly influence pubertal maturation. A combination of these two pathways may also exist, whereby neighborhood stressors and poor psychological functioning lead to emotional eating, reduced physical activity and overweight, which in turn may promote earlier puberty.

Past studies examining environmental determinants of pubertal timing have exhibited methodological limitations. One such limitation is that many studies have focused on menarche as an outcome, which occurs relatively late in the pubertal transition. Other studies have examined overall pubertal development without distinguishing between onset of breast development (thelarche) and onset of pubic hair (pubarche) development. Thelarche and pubarche represent observable markers of underlying hormones that are dependent on the maturation of unique endocrine axes, i.e., the hypothalamic-pituitary-gonadal (HPG) and hypothalamic-pituitary-adrenal (HPA) axis, respectively. Neighborhood factors might conceivably act on these hormonal pathways differentially. For instance, neighborhood conditions that lead to increases in body fat, which is associated with estrogen production, are likely to trigger the HPG axis resulting in accelerated breast development. Alternatively, environmental stress and resulting cortisol release (a measure of stress response) may awaken the HPA axis and accelerate pubic hair development [19,35]. As such, it is important to examine these early pubertal markers (thelarche and pubarche) separately in an effort to better understand potential hormonal responses to neighborhood environments. Moreover, these systems may play differential roles in the etiology of downstream health outcomes, such as breast cancer and other reproductive cancers.

In addition to the aforementioned limitations, studies of environmental determinants of pubertal onset have often been limited by study design. Many were cross-sectional, therefore limiting causal inference. Of those that were longitudinal, often participants were recruited after pubertal onset occurred, i.e., when girls were already peri-pubertal. As such, there is a need for longitudinal research that recruits girls at younger ages and follows them through the pubertal transition.

The current study aims to address these gaps in the extant literature. We utilized 5 waves of data from a larger ongoing study of ethnically-diverse girls and their parents/caregivers. Using direct observations of neighborhood environment and clinic-based Tanner stage assessments, we investigated the contribution of neighborhood factors to timing of onset of breast and pubic hair development, while adjusting for family income. Because girls and their families may be differentially affected by their neighborhoods depending on their contextual and cultural backgrounds, we also examined the modifying (moderating) effect of girls' ethnicity on the associations between neighborhood factors and pubertal outcomes. Moreover, in order to understand potential mechanisms, we investigated whether BMI operated in the causal pathway (i.e., mediated the relationship) between neighborhood factors and onset of puberty.

## Methods

### Participants and procedure

This project was carried out as part of the NIEHS/NCI Breast Cancer and the Environment Research Centers, four centers with transdisciplinary research collaborations across biologic, epidemiologic, and community outreach projects [36]. The present investigation focused on one epidemiologic project, the Cohort Study of Young Girls' Nutrition, Environment, and Transitions (CYGNET). The CYGNET study began in 2005 when 444 6-8-year-old girls and their primary caregivers (over 90% biological mothers) were recruited from the Kaiser Permanente Northern California (KPNC) health plan membership. Eligible members were identified through the KPNC Infant Cohort File, a database containing information on all live births occurring in KPNC facilities or to KPNC members. Details of the CYGNET study are described elsewhere [18,37]. Study procedures were approved by the Kaiser Permanente and UCSF Institutional Review Boards.

Participants for the current report (n = 213) were randomly sampled from the 444 participating families in the larger study. We focused on the first 5 data collection points (baseline and 4 annual follow-up visits) from this ongoing longitudinal study. Girls were 6-8 years old at baseline and 10-12 years old at last follow-up assessment for the current investigation. Intervals between participants' clinic visits were approximately one year, depending on the family's scheduling needs, and every effort was made to schedule within 1-2 months of one year. At baseline, informed assent and consent were obtained from participating girls and their caregivers, respectively. At each annual clinic visit, girls' anthropometric measurements and Tanner staging for breast and pubic hair development were assessed in clinic by trained researchers. Interviews were conducted with caregivers to collect additional information, including demographics. Neighborhood environment data were collected for this subsample of 213 participants through in-person street observation.

### Measures

#### Pubertal onset

Pubertal onset was assessed by clinical exam using the 5-stage Tanner staging system, which is widely utilized to describe the onset and progression of pubertal changes [38,39]. Assessment of Tanner stage for breast and pubic hair development was conducted at each annual clinic visit by rigorously trained research assistants under the supervision of a pediatric endocrinologist. Stage of breast development was assessed by visual inspection and palpation, and stage of pubic hair development was assessed by visual inspection. Onset of breast and pubic hair development were coded separately as: "no onset" (Stage 1) or "onset" (Stage 2 or above).

#### Neighborhood environment

Direct observations of girls' neighborhoods were conducted using a modified version of the St. Louis Audit Tool [40]. Audit tool items fit into five categories: land use environment, transportation environment, facilities, park or playground contents, and physical disorder. Street observers were provided a map with a circle representing a quarter-mile radius drawn around each girl's residence. Details of the direct observation method and resultant scales are described elsewhere [37]. A rigorous factor analysis resulted in five neighborhood scales: 1) mixed residential and commercial (included two-, three-, four-family homes ("walk-ups"); apartment building/complex; presence of sidewalks; place of worship; community center; day care or preschool; convenience or small grocery store); 2) food and retail (included chain fast food restaurant; supermarket; other convenience food restaurant; laundry or dry cleaners; full-service restaurant; coffee shop); 3) recreation (included park; walking or hiking trails; sports/playing field, basketball courts or tennis courts); 4) walkability (included street shoulders or wide outside lanes; curb bulb out/curb extension; traffic circle/roundabout); and 5) physical disorder (included garbage, litter, or broken glass in sidewalks or streets; graffiti on buildings) [37]. Cronbach's alphas for the neighborhood scales ranged from 0.50 to 0.87. A higher score in any of the indices indicated a greater presence of those attributes in a girl's neighborhood environment. For example a higher score in the 'food and retail' indices would indicate greater presence of fast food outlets, convenience stores and/or retail food outlets.

#### Ethnicity

Girl's ethnicity was assessed using primary caregiver's report at baseline and was coded as non-Hispanic White, African American or Black, Hispanic or Latino, Asian American, or Other.

#### Family income

Caregivers reported annual family income at baseline. Income categories were: < $25,000; $25,000-$49,999; $50,000-$74,999; $75,000-$99,999; ≥ $100,000. Income was dichotomized into "lower" (< $50 K/year) and "higher" (> $50 k/year) income.

#### Body mass index (BMI)

Height and weight measurements were obtained in clinic using calibrated scales and fixed stadiometers. Measurements at baseline were used to calculate BMI as weight (kg)/(height (m))^2^. BMI values were standardized for age and percentiles and z-scores calculated, using methods and standard distributions as provided by the Centers for Disease Control and Prevention, and BMI was treated continuously.

### Analysis

Data were analyzed using survival analysis with interval censoring in STATA version 11 using 5 data points (baseline and 4 annual follow-up visits). Weibull proportional hazard models were used to account for interval censoring, which adjusted for left and right censoring issues (given that some girls were pubertal at baseline and some were not pubertal by Year 5) and for censoring issues between visits (given that girls experienced pubertal onset at some unknown point between their annual visits). The effects of neighborhood factors on pubertal onset were examined for breast and pubic hair development, respectively, while adjusting for family income. Each neighborhood factor was examined separately. Interactive effects between neighborhood factors and ethnicity (Neighborhood Factor × Ethnicity) were included in each model separately. The mediating role of BMI was tested using the approach described by Baron and Kenny [41].

To control for hereditary factors, mother's age at menarche was considered as a covariate but was not significantly associated with either onset of breast (HR = 1.0 [0.97-1.03], *p *= 0.881) or pubic hair (HR = 1.0 [0.98-1.03], *p *= 0.795) development in bivariate analysis and remained non-significant even after adjusting for race and income. Therefore it was not included in subsequent analyses.

## Results

Girls were 7.4 years old on average at baseline and ethnically diverse (Table 1). Twenty-two percent of families had annual household incomes below $50,000/year, which reflects the high cost of living in the San Francisco Bay Area. Overall, 5% of girls had experienced onset of breast development at baseline, and 77% by the 4^th ^follow-up visit (Table 2). For pubic hair development, 7% and 69% had onset at baseline and by 4^th ^follow-up exam, respectively. Consistent with national data, African American girls were more likely to exhibit breast and pubic hair onset at baseline compared to other ethnic groups; 10% had experienced breast onset and 20% pubic hair onset. Asian girls were the least likely to have entered puberty at baseline and at 4^th ^follow-up exam. Average BMI was 17.2, and 30% of girls were "overweight" based on the CDC cutpoint (≥ 85^th ^percentile BMI by age). As expected, overweight girls were more likely to have experienced pubertal onset at younger ages compared to their non-overweight peers. Of those who were overweight at baseline (n = 64), 88% had breast onset and 78% pubic hair onset by the 4^th ^follow-up exam compared to 73% and 64% of non-overweight girls, respectively (Table 2).

**Table 1:** Descriptive characteristics of study participants (n = 213)

Characteristic Girls' Ethnicity	n	%
**Race/ethnicity**		

African American	51	23.9

Hispanic	50	23.5

Asian	23	10.8

White	88	41.3

Other	1	0.5

**Family income**		

< $50,000	46	21.6

≥ $50,000	167	78.4

**Girls' Overweight status (BMI ≥ 85th percentile for age)**		

Not overweight	149	70.0

Overweight	64	30.0

	mean	range

Age at baseline (in years)	7.4	6.5-8.1

Age of birth mother	40.0	22.0-54.0

Girls' weight in kg	27.2	16.8-57.3
	
Girls' body mass index (BMI)	17.23	12.2-29.0

**Table 2:** Breast and pubic hair onset at baseline and by follow-up 4 (n = 213)

		Number with breast/pubic hair onset			
		At baseline		At follow-up 4	
	**N**	**n**	**%**	**n**	**%**

**Breast Onset**	**213**	**11**	**5.2**	**164**	**77.0**

**By race/ethnicity**					

African American	51	5	9.8	45	88.2

Hispanic	50	4	8.0	40	80.0

Asian	23	0	0.0	18	78.3

White	88	2	2.3	61	69.3

Other	1	0	0.0	0	0.0

**By baseline weight**					

Not overweight	149	3	2.0	108	72.5

Overweight^a^	64	8	12.5	56	87.5

**Pubic hair onset**	**213**	**15**	**7.0**	**146**	**68.5**

**By race/ethnicity**					

African American	51	10	19.6	43	84.3

Hispanic	50	2	4.0	38	76.0

Asian	23	0	0.0	13	56.5

White	88	3	3.4	52	59.1

Other	1	0	0.0	0	0.0

**By baseline weight**					

Not overweight	149	6	4.0	96	64.4

Overweight^a^	64	9	14.1	50	78.1

^a^Based on ≥ 85^th ^percentile as established by the CDC

Unadjusted (bivariate) analyses (Table 3) indicated marginal associations between breast onset and "Mixed and Commercial Land Use" (HR = 1.04, *p *= .09), "Recreation" (HR = 0.9, *p *= .07) and "Disorder" (HR = 1.15, *p *= .08). Higher BMI (1.09, *p *< 0.01), African American ethnicity (HR = 2.53, *p *< 0.01) and low household income (HR = 1.82, *p *= 0.002) at baseline were significantly associated with increased rates of breast onset. "Recreation" was significantly associated with delayed onset of pubic hair (HR = 0.88, *p *= 0.03), while higher BMI (HR = 1.09, *p *< 0.01), low household income (HR = 2.10, *p *< 0.01), and African American ethnicity (HR = 2.99, *p *< 0.01) were associated with increased rates of pubic hair onset.

**Table 3:** Unadjusted, bivariate associations between neighborhood and demographic characteristics and pubertal onset (n = 213)

	Breast Onset		Pubic Hair Onset	
	Hazard Rate	*p*	Hazard Rate	*p*
**Neighborhood factors**				

Mixed land use	1.04	.09	1.02	.45

Food and retail	1.01	.70	0.98	.55

Recreation Outlets	0.90	.07	0.88	.03

Walkability	0.94	.34	0.94	.30

Disorder	1.15	.08	1.04	.60

Body Mass Index	1.09	< .01	1.09	< .01

Low income(< $50,000)	1.82	< .01	2.10	< .01

Ethnicity				

African American	2.53	< .01	2.99	< .01

Hispanic	1.33	.18	1.43	.10

Asian	1.22	.48	0.64	.16

Other	0.00	1.00		

White	ref		ref	

In multivariable analysis, after controlling for ethnicity and family income, only "Recreation" remained marginally associated with breast development (Table 4). When BMI was adjusted for in the model, Recreation was significantly associated with delayed breast onset (HR = 0.89, *p *= 0.03). There were no main effects for neighborhood factors with regard to onset of pubic hair development.

**Table 4:** Adjusted associations of neighborhood factors and pubertal outcomes (n = 213)

Neighborhood Variable	Before adjusting for BMI		After adjusting for BMI	
	HR(95% CI)	*p*-value	HR(95% CI)	*p*-value
**Breast onset**				

Mixed land use	1.01 (0.97-1.06)	0.54	1.01 (0.96-1.06)	0.67

BMI			1.09 (1.03-1.15)	< 0.01

Food and retail	1.01 (0.94-1.08)	0.87	1.01 (0.94-1.08)	0.75

BMI			1.09 (1.03-1.15)	< 0.01

Walkability	0.93 (0.82-1.06)	0.30	0.91 (0.8-1.04)	0.18

BMI			1.09 (1.04-1.15)	< 0.01

Recreation	0.91 (0.82-1.01)	0.09	**0.89 (0.79-0.99)**	**0.03**

BMI			1.1 (1.04-1.16)	< 0.01

Disorder	1.09 (0.91-1.29)	0.35	1.06 (0.89-1.27)	0.51

BMI			1.09 (1.03-1.15)	< 0.01

**Pubic hair onset**				

Mixed land use	0.98 (0.94-1.03)	0.49	0.98 (0.94-1.03)	0.54

BMI			1.08 (1.02-1.15)	0.01

Food and retail	0.97 (0.9-1.05)	0.43	0.98 (0.91-1.05)	0.54

BMI			1.08 (1.02-1.14)	0.01

Walkability	0.89 (0.78-1.02)	0.09	0.90 (0.78-1.03)	0.11

BMI			1.08 (1.02-1.14)	0.01

Recreation	0.89 (0.79-1.01)	0.06	0.91 (0.81-1.03)	0.12

BMI			1.08 (1.02-1.14)	0.01

Disorder	0.97 (0.82-1.15)	0.72	0.95 (0.8-1.14)	0.60

BMI			1.08 (1.03-1.15)	< 0.01

All models were adjusted for ethnicity and income

In multivariable analyses including neighborhood factor × ethnicity interaction terms, Recreation × Ethnicity was significant for both timing of breast and pubic hair development (Table 5). Interaction terms for other neighborhood factors were not significant. After adjusting for BMI, stratified analyses (Table 6) showed that Recreation was significantly associated with timing of breast onset and pubic hair onset for African American girls only. There were no significant effects in other ethnic groups.

**Table 5:** Adjusted associations between Recreation and pubertal outcomes, including Recreation × ethnicity interaction term (n = 213)

	Breast Onset			Pubic Hair Onset		
**Variable**	**Coef**	**HR**	***p*-value**	**Coef**	**HR**	***p*-value**

Recreation	0.05	1.05	0.611	0.09	1.09	0.387

Low income(< $50,000)	0.38	1.46	0.137	0.64	1.90	0.012

BMI	0.10	1.11	0.000	0.08	1.08	0.006

Ethnicity						

*African American*	1.17	3.23	0.000	1.50	4.49	0.000

*Hispanic*	0.20	1.22	0.533	0.00	1.00	0.995

*Asian*	0.52	1.68	0.146	-0.01	0.99	0.986

*White*	ref			ref		

Recreation *× African American*	-0.33	**0.72**	**0.035**	-0.44	**0.64**	**0.010**

Recreation *× Hispanic*	-0.23	0.80	0.127	-0.11	0.89	0.459

Recreation *× Asian*	-0.15	0.86	0.396	-0.36	0.70	0.126

Recreation *× White*	ref			ref		

**Table 6:** Adjusted associations of Recreation and pubertal outcomes by race (n = 213)

Subgroup	Before adjusting for BMI		After adjusting for BMI	
	HR(95% CI)	p-value	HR(95% CI)	p-value
**Breast onset**				

African American	0.83(0.65-1.04)	0.106	0.74(0.56-0.97)	**0.032**

Hispanic	0.86(0.68-1.09)	0.208	0.87(0.68-1.10)	0.229

Asian	0.92(0.68-1.24)	0.584	0.88(0.66-1.18)	0.402

White	1.07(0.88-1.30)	0.488	1.06(0.88-1.29)	0.534

**Pubic hair onset**				

African American	0.75(0.58-0.96)	**0.022**	0.72(0.55-0.94)	**0.015**

Hispanic	0.84(0.67-1.05)	0.125	0.86(0.68-1.09)	0.217

Asian	0.75(0.49-1.15)	0.190	0.74(0.47-1.16)	0.189

White	1.10(0.90-1.34)	0.368	1.10(0.90-1.34)	0.364

All models were adjusted for income

To test the potential mediating role of BMI in the relationship between Recreation and pubertal outcomes for African Americans, we first examined the direct association between Recreation and BMI by ethnic group (Table 7). After controlling for family income, Recreation was not significantly associated with BMI, indicating that BMI was not in the causal path between Recreation and pubertal onset. We also compared the effects of Recreation on breast and pubic hair onset with and without adjusting for BMI. The effects of Recreation on the two pubertal outcomes persisted when BMI was included in the models.

**Table 7:** Association between neighborhood factors and BMI by Ethnicity (n = 213)

	African American		Hispanic		Asian		White	
	**Coef**	***p*-value**	**Coef**	***p*-value**	**Coef**	***p*-value**	**Coef**	***p*-value**

Mixed land use	0.13	0.34	0.01	0.94	-0.05	0.58	0.01	0.84

Food and retail	0.36	0.12	-0.01	0.97	-0.07	0.47	-0.06	0.57

Recreation	0.05	0.90	-0.31	0.43	0.07	0.74	-0.07	0.66

Walkability	-0.16	0.74	0.20	0.63	-0.34	0.30	0.08	0.61

Disorder	1.22	0.04	-0.50	0.43	-0.24	0.44	0.37	0.06

All models were adjusted for income

## Discussion

This investigation examined the influence of girls' neighborhood environments on their timing of onset of breast and pubic hair development, while taking into consideration BMI, family income and ethnicity. Consistent with past studies, African American girls, those from lower income families, and girls with higher BMI were at greatest risk for experiencing breast development at younger ages. Asian girls and those with lower BMI were at reduced risk for experiencing pubertal onset at younger ages.

We found that African American girls who lived in neighborhoods characterized as having more recreational outlets (Recreation) experienced lower rates of onset of breast and pubic hair development over the 4 years of follow-up of our study, i.e., by ages 10-12 years. For each one-unit increase in the neighborhood Recreation index, African American girls experienced a 26% and 28% decrease in hazard rates for breast and pubic hair onset, respectively. The Recreation index included neighborhood factors such as availability of parks, walking or hiking trails, playing fields, and basketball or tennis courts. These results suggest that physical activity may play a key role in determining accelerated onset of breast and pubic hair development among young African American girls. There were no significant neighborhood effects on pubertal development among girls from other ethnic groups. Therefore, the availability of recreational outlets may be particularly important for African Americans. Further investigation to better understand these ethnic-specific effects is warranted.

Being overweight is a well-established risk factor for early entrance into puberty, and hormonal mechanisms related to adiposity are likely to explain the relationship between BMI and pubertal acceleration [9,15,34]. Although girls' overweight was strongly predictive of timing of pubertal onset in our sample, we did not find evidence that BMI mediated the association (was in the causal pathway) between the recreational environment and pubertal timing for African American girls. In fact, there was no significant association between Recreation and BMI, neither in bivariate (unadjusted) tests nor after adjusting for family income. Rather, the effect of Recreation on pubertal outcomes was independent of the effect of BMI. A number of rigorous epidemiological studies, however, have found direct associations between neighborhood availability of recreational outlets and overweight. For example, a recent study using data from the National Survey of Children's Health, found that children living in environments with no parks, playgrounds, recreation or community centers were at higher odds for experiencing obesity, and that neighborhood effects were more pronounced among younger children (10-11 years old) and among girls [30]. This study also illustrated clear ethnic and socioeconomic disparities in overweight among children, with ethnic minority youth and those in poverty showing the highest rates of obesity and overweight [30]. It is likely that these girls were at elevated risk for early puberty as well, although pubertal outcomes were not investigated. Other studies have also shown marked disparities in neighborhood access to physical activity facilities, with lower socioeconomic and high-density minority neighborhoods having fewer recreational outlets, and residents in these neighborhoods, in turn, exhibiting decreased physical activity levels and increased overweight [32].

Our findings provide preliminary evidence that neighborhood availability of recreational outlets, which signal opportunities for increased engagement in physical activity and reduced sedentary activity, influence African American girls' pubertal timing. Although body composition assessed using BMI is clearly an important determinant of pubertal timing, physical activity levels and/or sedentary behaviors may act as more proximal, and potentially modifiable, explanatory factors that influence the relationship between neighborhood environment and pubertal timing. As such, neighborhood recreation outlets may serve as important protective factors for young African American girls during this developmental period. Future studies should delve deeper to assess girls' utilization of neighborhood recreational facilities, and their relative activity levels, to determine whether subjective and objective measures of physical activity account for the relationship between availability of recreation facilities and timing of breast development. It may also be important to consider whether availability of recreational outlets and physical activity levels interact with African American girls' overweight to influence pubertal onset (e.g., are effects greater for overweight girls?). Finally, our findings suggest that further exploration of hormonal mechanisms is critical in order to better understand whether physical activity might counteract or ameliorate the accelerating effects of overweight and adiposity on girls' pubertal development.

### Limitations

This investigation included data from the first five annual clinic visits of an ongoing prospective study. At 4^th ^follow-up, 23% of girls had not yet experienced breast onset, and about 32% had not experienced onset of pubic hair development. As a result, we were not able to capture later pubertal onset (or delayed puberty) nor were girls old enough to allow for the examination of menarche. We were also unable to assess the rate at which girls progressed through puberty, also known as pubertal tempo, given the girls' ages. As girls in the sample grow older, we will be able to examine these outcomes. Neighborhood characteristics were defined geographically, based on audits conducted within a quarter-mile radius of the block containing the girls' residences. While findings showed significant effects of the recreational environment immediately surrounding a girl's residence, the environment that a girl may frequent outside of this circumscribed area (e.g., access to recreation in the school environment) was not accounted for in this investigation and may have contributed to the ethnic differences in our findings. Moreover, the relatively small sample size may have limited our power to detect interactions between ethnicity and other neighborhood factors; however, this also lends confidence to our significant interactive findings for African American girls. Finally, other contextual and familial factors that are known to influence pubertal timing, such as family composition and childhood sexual abuse, were beyond the scope of the current study but are important determinants of physical health and mental health over the life course.

This study had considerable strengths. Tanner stage assessments for pubertal onset were conducted using clinical examination by trained clinic staff based on standardized methods, which is considered the gold standard in the field [13]. Potential misclassification of breast development due to adiposity was minimized in this study as palpation and visual inspection were both utilized to assess onset of breast development. Assessment of height and weight to calculate BMI was conducted in clinic using standardized and reliable measurement tools. In addition, we employed direct in-person observations to assess neighborhood environments rather than relying on aggregate measures such as those available from census tract data.

## Conclusions

This is the first known study to prospectively examine the effects of a girl's neighborhood environment on her pubertal timing. As such, this study represents an initial step towards understanding the potentially complex effects of various neighborhood level factors on pubertal maturation. Results demonstrated that availability of recreational outlets was significantly related to timing of breast and pubic hair development for African American girls, even when strong predictors of puberty such as income and BMI were considered. While all normally-developing girls experience puberty, early timing of pubertal onset contributes to both short-term health consequences in adolescence and risk for longer-term negative health outcomes among women, including obesity, breast and other reproductive cancers, and cardiovascular disease. Moreover, physical activity is a known protective factor for many of these health outcomes in adulthood [42-44]. As such, a better understanding of how neighborhood factors, particularly opportunities for physical activity, influence girls' pubertal timing may inform successful intervention strategies and policy development to promote better health over the life course for women.

## Abbreviations

BMI: Body mass index; HPG axis: Hypothalamic-pituitary-gonadal axis; HPA axis: Hypothalamic-pituitary-adrenal axis; NIEHS: National institute of environmental health sciences; NCI: National cancer institute; CYGNET: Cohort study of young girls' nutrition, environment, and transitions; KPNC: Kaiser permanente northern california

## Competing interests

The authors declare that they have no competing interests.

## Authors' contributions

JD took the lead in conceptualizing this paper, was involved in pubertal data collection and clinic interviews, developed the analytic plan, interpreted the findings and wrote the majority of the paper. MF conducted the literature review, helped conceptualize the study, conducted preliminary analyses, and drafted portions of the paper. JPE conducted the majority of the analyses, contributed to writing the results section and created the tables. LK designed the study, oversaw collection of pubertal and demographic data, contributed conceptually to the paper, and provided edits. LG oversaw the Tanner staging methodology, ensured the quality of the pubertal data, contributed conceptually to the paper, and provided edits. IY conceived the study, contributed to study design, oversaw collection of the neighborhood data, contributed conceptually to this paper, and drafted portions of the paper. All authors read and approved the final manuscript.

## Pre-publication history

The pre-publication history for this paper can be accessed here:

http://www.biomedcentral.com/1471-2431/12/27/prepub
